# Salidroside ameliorates cerebral ischemic injury and regulates the glutamate metabolism pathway in astrocytes

**DOI:** 10.3389/fphar.2024.1472100

**Published:** 2024-11-12

**Authors:** Xiaoyu Zheng, Hongwei Zhang, Yehao Zhang, Zhao Ding, Zishan Huang, Haoran Li, Mingjiang Yao, Wenting Song, Jianxun Liu

**Affiliations:** ^1^ Beijing Key Laboratory of Traditional Chinese Medicine Pharmacology, Xiyuan Hospital, Chinese Academy of Traditional Chinese Medicine, National Research Center for Clinical Medicine of Cardiovascular Diseases of Traditional Chinese Medicine, Beijing, China; ^2^ National Clinical Research Center for Chinese Medicine Cardiology, Xiyuan Hospital, Chinese Academy of Traditional Chinese Medicine, Beijing, China; ^3^ Research Institute of Traditional Chinese Medicine, Guangdong Pharmaceutical University, Guangzhou, China

**Keywords:** salidroside, cerebral ischemia, astrocyte, glutamate metabolism, glutamine synthetase, glutamate transporter 1

## Abstract

**Background and Aim:**

Salidroside (SA) is the main active component of *Rhodiola rosea* L., with potential in treating cardiovascular and cerebrovascular diseases and cerebral ischemia. However, its efficacy and mechanism in cerebral ischemia remain unclear, particularly regarding its effect on glutamate (Glu) metabolism. In this paper, we aimed to investigate the efficacy of SA in treating cerebral ischemia and its pharmacological mechanism.

**Experimental procedure:**

We studied the effects of SA on SD rats with cerebral ischemia, evaluating neurobehavior, cerebral water content, infarct size, and brain microstructure. We also assessed its impact on glial fibrillary acidic protein (GFAP), glutamine synthetase (GS), and glutamate transporter 1 (GLT-1) proteins using immunohistochemistry and Western blot. Additionally, we used SVGp12 cells to simulate cerebral ischemia and measured Glu levels and used Western blot to observe the level of GS and GLT-1.

**Results:**

SA improved neural function, reduced infarct size, and regulated GSH and Glu levels in rats. In cell experiments, SA increased cell viability and decreased Glu concentration after ischemia induction. It also regulated the expression of GFAP, GS, and GLT-1.

**Conclusion:**

SA alleviates cerebral ischemia-induced injury by acting on astrocytes, possibly through regulating the glutamate metabolic pathway.

## Graphical Abstract 1

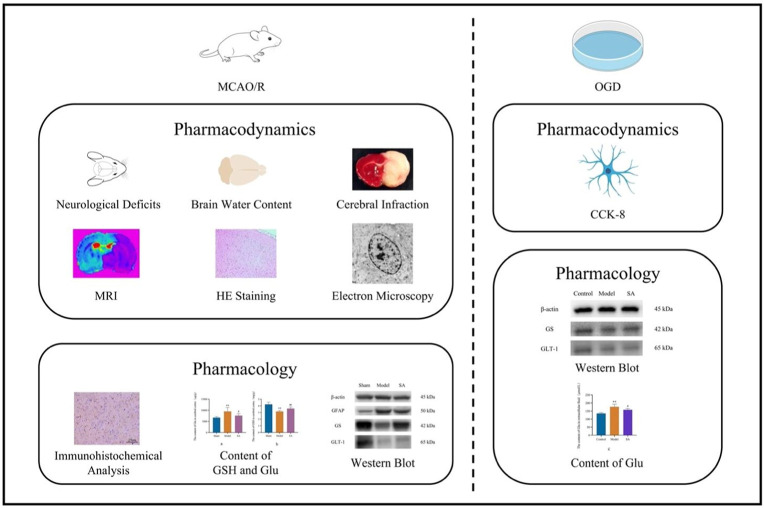

## Graphical Abstract 2

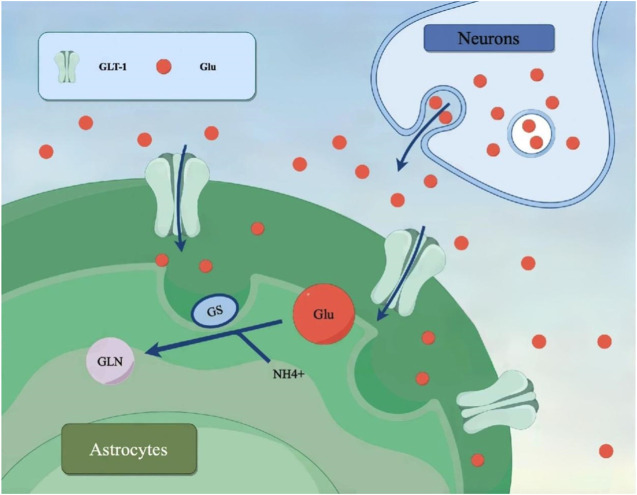

## Highlights

• From the perspective of studying astrocyte swelling, this study observed that salidroside (SA) inhibited cerebral edema after cerebral ischemia by inhibiting astrocyte swelling.
• SA can act on astrocytes, regulate the pathway of glutamate (Glu) metabolism, and improve the cytotoxicity after cerebral ischemia.
• The conclusions of this paper have been mutually verified by cell and animal experiments.

## 1 Introduction

Stroke is a condition that causes brain tissue damage due to the sudden rupture of blood vessels in the brain or blockage of blood vessels that prevents blood from flowing into the brain, with high mortality and high disability rates. According to the different pathogenesis and pathological basis, stroke can be divided into ischemic stroke and hemorrhagic stroke. Ischemic stroke after cerebral artery occlusion is one of the major causes of chronic disability worldwide (Zhu et al., 2022). In contemporary society, ischemic stroke accounts for approximately 85% of the incidence of stroke (Beal, 2010) and has become a major disease threatening human health. Cerebral ischemia often leads to brain edema, which is mainly divided into vasogenic and cytotoxic brain edema. Vasogenic brain edema occurs due to the breakdown of the tight connections between vascular endothelial cells, which increases the permeability of the blood–brain barrier (BBB), and a large amount of blood fluid enters the extracellular space of the brain parenchyma, leading to the breakdown of the BBB. In addition, cytotoxic brain edema occurs when the BBB is intact, mainly due to astrocyte metabolic disorder, which affects the function of the ion pump on the membrane and triggers a large number of water molecules to enter the cells, resulting in cellular edema (Nag et al., 2009).

Astrocytes are the most abundant cells in the central nervous system (CNS). They have a strong tolerance when cerebral ischemia occurs and can protect neurons through various ways to reduce cerebral ischemia reperfusion injury (Koizumi et al., 2018). When cerebral ischemia occurs, astrocytes undergo changes such as cell hypertrophy, cell swelling, increased prominences, and proliferate in and around the ischemic site. Under normal physiological conditions, astrocytes have functions such as integrating nerve signals, inhibiting Ca^2+^ excitation, processing information, and bridging neurons and vascular endothelial cells (Perea et al., 2009). Their membrane and cytoplasm have a variety of ion channels, receptors, high-affinity carriers of neuroactive amino acids, and enzymes to maintain the balance of the internal environment (Clément et al., 2020). Astrocytes can also produce various regulatory signals, synthesize neurotrophic mediators, and reactivate and metabolize glutamate (Glu), thereby protecting neurons (Yu and Lau, 2000). Therefore, maintaining the stability of astrocyte morphology and function plays an important role in improving the neuronal injury caused by cerebral ischemia.

Glu is the most common excitatory neurotransmitter in the CNS. During cerebral ischemia, the level of Glu increases significantly, leading to Glu-induced neuronal toxicity, which is thought to be the initial trigger of ischemic injury (Kostandy, 2012). Glutamate transporter 1 (GLT-1, also known as EAAT2) on astrocytes is responsible for the majority of extracellular Glu clearance and is essential for preventing excitatory toxicity in the brain (Zhang et al., 2019). The upregulation of GLT-1 shows a beneficial effect on ischemia-induced neuronal damage. In addition, Glu transported to astrocytes is further degraded by glutamine synthetase (GS), and the downregulation of GS increases glutamate toxicity and induces severe neuronal damage (Rose et al., 2013). Both GLT-1 and GS exist in brain astrocytes and are expressed in large quantities, and GLT-1 and GS are the main pathways to process and clear Glu.

**FIGURE 1:**
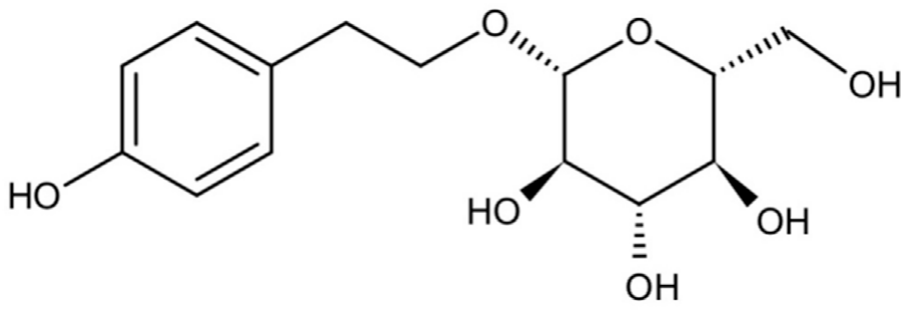
Chemical structure of salidroside.

*Rhodiola rosea* L. is a traditional Chinese medicine. The dried roots and rhizomes of the Rhodiola plant are used as medicine. Its main effect is “tonifying qi, promoting blood circulation, and relieving asthma.” Salidroside (SA) is the main active component of Rhodiola and is a type of phenylpropanoid glycoside. Studies have found that SA has pharmacological effects, such as anti-inflammatory, anti-apoptosis, anti-hypoxia, anti-depression, and anti-oxidation (Zhong et al., 2018; Fan et al., 2020), and is clinically used in the treatment of Alzheimer’s disease, Parkinson’s disease, epilepsy, cancer, diabetes, liver injury, and other diseases (Fan et al., 2020; Zhong et al., 2018; Wang et al., 2019; Liu et al., 2018). A number of studies have shown that SA can protect neuronal damage caused by cerebral ischemia both *in vivo* and *in vitro* (Chen et al., 2016; Zhang et al., 2020). However, due to the multi-component, multi-level, and multi-target action characteristics of traditional Chinese medicine (TCM), the mechanism of SA in the treatment of cerebral ischemia has not been fully explored. Previous studies have focused on the protective effects of SA on neurons and vascular endothelial cells after ischemia. However, astrocytes are the most widely distributed cells in the brain and participate in the formation of the BBB, secreting a variety of neurotransmitters and cytokines. Astrocytes can protect against neuronal injury caused by cerebral ischemia through multiple mechanisms (Hinkle et al., 2019), such as the uptake of excitatory amino acids (Lau et al., 2010), inhibition of the release of inflammatory mediators (Regunathan and Piletz, 2003), enhancement of gap junctions (Gawdi et al., 2023), anti-oxidation (Pekny and Nilsson, 2005), and inhibition of apoptosis (Pei et al., 2019). Therefore, the study of the regulatory effect of SA on astrocytes after cerebral ischemia can more comprehensively explain the mechanism of salidroside brain protection. This study focused on the regulation and improvement of SA on astrocytes in the rat brain after cerebral ischemia and observed and analyzed the expression of GS and GLT-1 proteins distributed on astrocytes, which is the uniqueness of this study. The chemical structure of SA is shown in Figure 1.

## 2 Experimental procedures

### 2.1 Experimental animals and drug administration

Male Sprague–Dawley (SD) rats weighing 210─230 g (approximately 7–8 weeks old) were used in the experiment. The rats were kept in a 12 h light/dark cycle, at a temperature of 23°C ± 1°C and a humidity of 55%± 5%, with free access to food and water. All rats used in the experiment were divided into four groups of 20 rats each, following the principle of minimizing animal harm. The rats were raised in the SPF Laboratory Animal Center of Xiyuan Hospital, China Academy of Chinese Medical Sciences, and the experimental operations fully complied with the ethical standards of the institution and were reviewed by the Ethics Committee of Xiyuan Hospital, China Academy of Chinese Medical Sciences (Ethics No. 2018XLC003-1). The research was conducted in accordance with the internationally accepted principles for laboratory animal use and care.

The groups included the normal group, the model group, the SA high-dose (SA-H) group, and the SA low-dose (SA-L) group. After 24 h of cerebral ischemia, rats that failed to develop the model or had died were excluded from the analysis. From each group, eight rats were randomly selected for assessment of neurological behavior, brain index, brain water content, and infarct area measurement. Additionally, six randomly selected rats from each group were subjected to histological examination using hematoxylin–eosin (HE) staining, immunohistochemical staining, immunoblotting, and analysis of glutathione (GSH), and Glu levels.

The dosage selection for SA was based on previous orthogonal experiments and validation experiments conducted in our laboratory. The SA-H group received a dosage of 2.5 mg·kg^−1^, while the SA-L group received 1.25 mg·kg^−1^. Administration was performed via intravenous tail vein injection. The drug was given immediately after cerebral ischemia and injected once within 24 h. The normal and model groups were administered an equivalent volume of physiological saline. All groups had access to normal food and water. For mechanistic studies, the SA-L group was utilized as the dosing group.

### 2.2 Transient middle cerebral artery occlusion model in rats

The rats underwent middle cerebral artery occlusion (MCAO) modeling (Zhou et al., 2021). This method is well-established in our laboratory. Anesthesia was induced by intraperitoneal injection of 4% chloral hydrate. The neck area was depilated and disinfected, and a midline incision was made. The right common carotid artery, external carotid artery, and internal carotid artery were separated. A microvascular clamp was used to occlude the internal carotid artery, and ligations were made at the proximal end of the common carotid artery and the external carotid artery, with a 2 mm distance from the bifurcation of the common carotid artery. A filament was inserted into the internal carotid artery and lightly secured in the far proximal end of the common carotid artery. The filament had a diameter of 0.24 mm and a head diameter of (0.32 ± 0.02) mm. The insertion depth was measured from the site of the vessel bifurcation, and it was secured at a depth of 2 cm within the most proximal end of the common carotid artery. Reperfusion was initiated 90 min after filament insertion, allowing blood to flow back into the cerebral arteries through the circle of Willis, achieving cerebral vascular reperfusion. The sham-operated group of rats underwent the same surgical procedure as the model group, except that no filament occlusion of the middle cerebral artery was performed. Finally, the surgical wounds were sutured, and the animals were returned to their cages, provided with regular access to water and food, and observed for their postoperative status.

### 2.3 Evaluation of neurological deficits

The rats were assessed at 6 and 24 h post-cerebral ischemia using the Longa 5-point scoring method, following the principles of randomization and blinding. The scoring criteria were as follows: 0 points, normal, no neurological deficits observed; 1 point, animals unable to fully extend the left forelimb; 2 points, paralysis of the left limb, circling to the left while walking, and tail chasing behavior; 3 points, animals falling to the left while walking, or inability to stand or roll; 4 points, lack of spontaneous movement and evidence of impaired consciousness. Neurological deficit scores within the range of 1–3 were considered indicative of successful modeling. The rats in all groups, except for the sham-operated group, were excluded if they scored 0 points (indicating unsuccessful modeling) or 4 points (indicating severe injury leading to death within 24 h). The rats were graded by an independent observer who was not involved in the experiment.

### 2.4 Measurement of cerebral water content

At 24 h post-MCAO surgery in rats, anesthesia was induced with an intraperitoneal injection of 4% chloral hydrate, followed by rapid decapitation. The brain was excised following cranial removal, and superficial blood and cerebrospinal fluid were gently removed using filter paper. The brain tissue was then placed in a pre-weighed culture dish, and its wet weight was determined using an electronic balance (precise to 0.001 g). Subsequently, the brain tissue was subjected to drying in a 60°C oven for 72 h until a constant weight was achieved, and the dry weight was measured. Brain tissue water content was calculated using the formula: Cerebral water content = (wet weight − dry weight)/wet weight × 100%.

### 2.5 Measurement of cerebral infarction

The rat skull was opened to access the brain, and surface blood and cerebrospinal fluid were gently absorbed using filter paper. The brain was rapidly sectioned into five slices along the coronal plane. These brain sections were immediately stained with a 1% solution of 2,3,5-triphenyltetrazolium chloride (TTC). Images capturing the distribution of ischemic lesions on the brain slices were acquired. Image Pro Plus (Media Cybernetics Inc.) software was utilized to outline the ischemic area and the total brain slice area for each slice, facilitating the calculation of the cerebral infarct area. The cerebral infarct area was determined using the formula: Cerebral infarct area = (infarct area/total brain slice area) × 100%.

### 2.6 Magnetic resonance imaging examinations

Magnetic resonance imaging (MRI) examinations were conducted at the Small Animal MRI Laboratory of the Institute of Materia Medica, Chinese Academy of Medical Sciences. A Bruker 8.0T MRI (Burker company), PharmaScan 70/16, United States, provided by the laboratory, was utilized for imaging. After 24 h of cerebral ischemia, rats were placed in an animal-specific anesthetic chamber, and the concentration of isoflurane was adjusted for inhalational anesthesia. The rats were positioned in a prone posture with their teeth secured in a stabilizing device, ensuring unobstructed respiration. The head was aligned in the center of the MRI coil for imaging.

First, a spin-echo sequence scan was performed to obtain T2-weighted imaging (T2WI) images with the following parameters: field of view (FOV) = 35 mm, echo time (TE) = 30 ms, matrix size (MTX) = 256 × 256, repetition time (TR) = 3.0 s, and slice thickness (ST) = 0.65 mm, consisting of a total of 30 layers. The FOV for each layer was 35 mm × 35 mm. Subsequently, diffusion-weighted imaging (DWI) sequences were acquired in the coronal plane. A single-shot echo-planar sequence was employed for measuring the apparent diffusion coefficient (ADC). The ADC value is one of the most sensitive parameters for assessing the degree of water diffusion integrity. The acquisition parameters for DWI were as follows: TR = 20 ms, TE = 2000 ms, MTX = 64 × 64, ST = 0.56 mm, and FOV = 35 mm × 35 mm, with diffusion encoding in 6 directions and a b-value (b represents the diffusion weighting factor) of 200 s/mm^2^. Finally, functional magnetic resonance imaging processing was conducted. T2WI and DWI images were imported into Paravision version 5.1 software (Kitware Corporation) configured in the Bruker 8.0 T MRI system. Subsequent image processing was carried out on both T2WI and DWI images.

### 2.7 HE staining

The rat brain was then sectioned into three pieces, and the central piece was selected. It was fixed in 4% tissue fixative, embedded in paraffin, and subjected to routine HE staining. The paraffin-embedded sections were deparaffinized in water, immersed in hematoxylin staining solution for 3–5 min, rinsed with tap water, subjected to differentiation, followed by bluing, and rinsed with running water. Subsequently, the sections were dehydrated through a graded alcohol series of 85% and 95%, stained with eosin for 5 min, dehydrated, mounted, and examined under a microscope for image capture and analysis.

### 2.8 Electron microscopy

At 24 h after cerebral ischemia, cerebral cortex slices measuring 1 cubic millimeter were crafted into square pieces with a vertex at the center of the ischemic area comprising part of the penumbra and undamaged area. These pieces were fixed for 2 h with 2.5% glutaraldehyde in PBS at room temperature. The pieces were washed with PBS, incubated for 1 h in a PBS solution containing 1% OsO_4_, dehydrated with ethanol, contrast-stained with 1% uranyl acetate, and embedded in EPON resin. Ultrathin sections were prepared and observed using a Leica EM UC6 ultramicrotome (Leica Geosystems, Switzerland) and a HITACHI H-7500 transmission electron microscope (HITACHI, Japan), respectively.

### 2.9 Immunohistochemical analysis

Routine paraffin sections were used to observe the protein expression of glial fibrillary acidic protein (GFAP), GS, and GLT-1 in brain tissue using immunohistochemistry. The basic method was as follows. The sections were deparaffinized, rinsed with PBS for 5 min × 3 times, subjected to microwave antigen retrieval for 20 min, again rinsed with PBS for 5 min × 3 times, treated with hydrogen peroxide for 30 min, and rinsed with PBS for 5 min × 3 times. Then, 50 μL of synapsin-Ⅰ, PSD-95, or α-synuclein antibody (1:50) was added dropwise and incubated for 2 h in a dark humidified box (37°C). After rinsing with PBS for 5 min × 3 times, 50 μL of goat anti-rabbit IgG was added dropwise and incubated in a dark humidified box (37°C) for 2 h. The sections were rinsed with PBS for 5 min × 3 times, and color development was controlled with DAB (1:50), followed by washing with distilled water to terminate color development. Hematoxylin counterstaining, routine dehydration, xylene transparency, and neutral gum sealing were performed. The stained sections were observed using an Olympus BX51 microscope (upright), and three fields of view were selected around the ischemic area of the ischemic cortex at a magnification of 20 × 10. An Olympus E330 digital camera (Olympus Corporation, Japan) was used to collect images, and Image-pro Plus software was used to analyze the immunohistochemical results.

### 2.10 Contents of GSH and Glu in ischemic brain tissue

First, the ischemic cerebral cortex of the rats was separated, and a lysis solution was added. The tissue was then lysed with a special extraction solution on ice and allowed to stand for precipitation. Then, centrifugation was performed for 10 min at 8,000 r·min^−1^ and 4°C, and the supernatant was collected for testing. Detection was carried out according to the instructions provided in the GSH and Glu content detection kit.

### 2.11 Culture of astrocytes and oxygen–glucose deprivation management and treatment

SVGp12 cells were thawed and resuspended and cultured in a normal culture medium (containing 10% fetal calf serum, 100 mg·L^−1^ penicillin, and 100 mg·L^−1^ streptomycin). culture conditions were 5% CO_2_ and 37°C. After being passed twice at a ratio of 1:3, all cells were collected by trypsin digestion, adjusted to a cell concentration of 1 × 10^5^ cells/mL, and seeded into a 96-well plate at a cell suspension volume of 100 μL per well. The cells could be used for experiments after 24 h of cultivation in the incubator.

The SVGp12 cells in the control group were replaced with 100 μL/well of normal culture medium, and each administration group was given different doses of 100 μL/well of drug-containing normal culture medium. Salidroside was dissolved in DMSO and diluted to the target concentration before administration. After 24 h, CCK-8 solution was added to determine the toxicity of salidroside on SVGp12 cells.

The SVGp12 cells in the control group were replaced with 100 μL/well of normal medium, while the model group was replaced with 100 μL/well of sugar-free medium, and each drug group was given different doses of 100 μL/well of a drug-containing sugar-free medium. Salidroside was dissolved in DMSO, diluted to the target concentration, and administered at the same time as oxygen–glucose deprivation/reoxygenation (OGD). After 4 h, oxygen, and glucose were restored, CCK-8 solution was added, and after 2 h, the effect of salidroside on OGD/R-induced SVGp12 cells was measured.

### 2.12 Determination of cell viability

The cells were seeded in a 96-well culture plate at a concentration of 1 × 10^5^ cells/mL and were used for experiments after 24 h of culture. The drug dose for measuring the toxicity of SA was reduced from 100 μmol·L^−1^, and the dose for measuring its efficacy was determined based on the toxicity results. SA was dissolved in DMSO.

Serum-free DMEM was added to the control group, and appropriate concentrations of DMSO or drugs were added to the remaining groups. After 4 h, oxygen, and glucose were restored; 10 μL of CCK-8 solution was added to each well and incubated in the incubator for 2 h; the absorbance was detected at a wavelength of 450 nm using a microplate reader; and the optimal dosage of the drug was determined.

### 2.13 Western blotting

The protein from SVGp12 cells and tissues containing ischemic penumbra was harvested at 24 h after cerebral ischemia, extracted with RIPA buffer (Beyotime Biotechnology, Shanghai, China), and mixed with protease and phosphatase inhibitor cocktails (MCE, NJ, United States). The protein concentration was determined using a protein assay solution (Epizyme Biotech, China). Identical quantities of protein were denatured using protein loading buffer (Solarbio, China), loaded onto 10% SDS–PAGE gels, and transferred to polyvinylidene difluoride (PVDF) membranes (MILLIPORE, United States) by electroblotting. The PVDF membranes were blocked by 5% bovine serum albumin (BSA) (Sigma-Aldrich, China) in Tris-buffered saline with Tween-20 (TBST) (Solarbio, China) buffer for 1 h and were incubated overnight at 4°C using the following antibodies: GFAP (Proteintech, China, 1:2,000), GS (Proteintech, China, 1:2,000), EAAT2 (Abcam, China, 1:2,000), and β-actin (Sigma, China, 1:5,000). Reactive bands were detected using an ECL detection reagent (Thermo Fisher Scientific, MA, United States) following the manufacturer’s instructions. The protocols for cell culture experiments were the same as those described above.

### 2.14 Statistical analysis

All data are presented as the mean ± SEM. The Shapiro−Wilk normality test was used to confirm whether the values followed a Gaussian distribution. Assumptions of equal variance were tested using Brown–Forsythe tests. Statistical significance was determined using the two-tailed unpaired Student’s t-test for two groups and one-way analysis of variance (ANOVA) followed by Tamhane’s post-hoc test or two-way ANOVA accompanied by Sidak’s test for multiple comparisons. In all cases, *p* < 0.05 was considered statistically significant. All statistical data were analyzed using GraphPad Prism software (San Diego, CA, United States).

## 3 Results

### 3.1 Salidroside improves brain damage caused by cerebral ischemia

**FIGURE 2:**
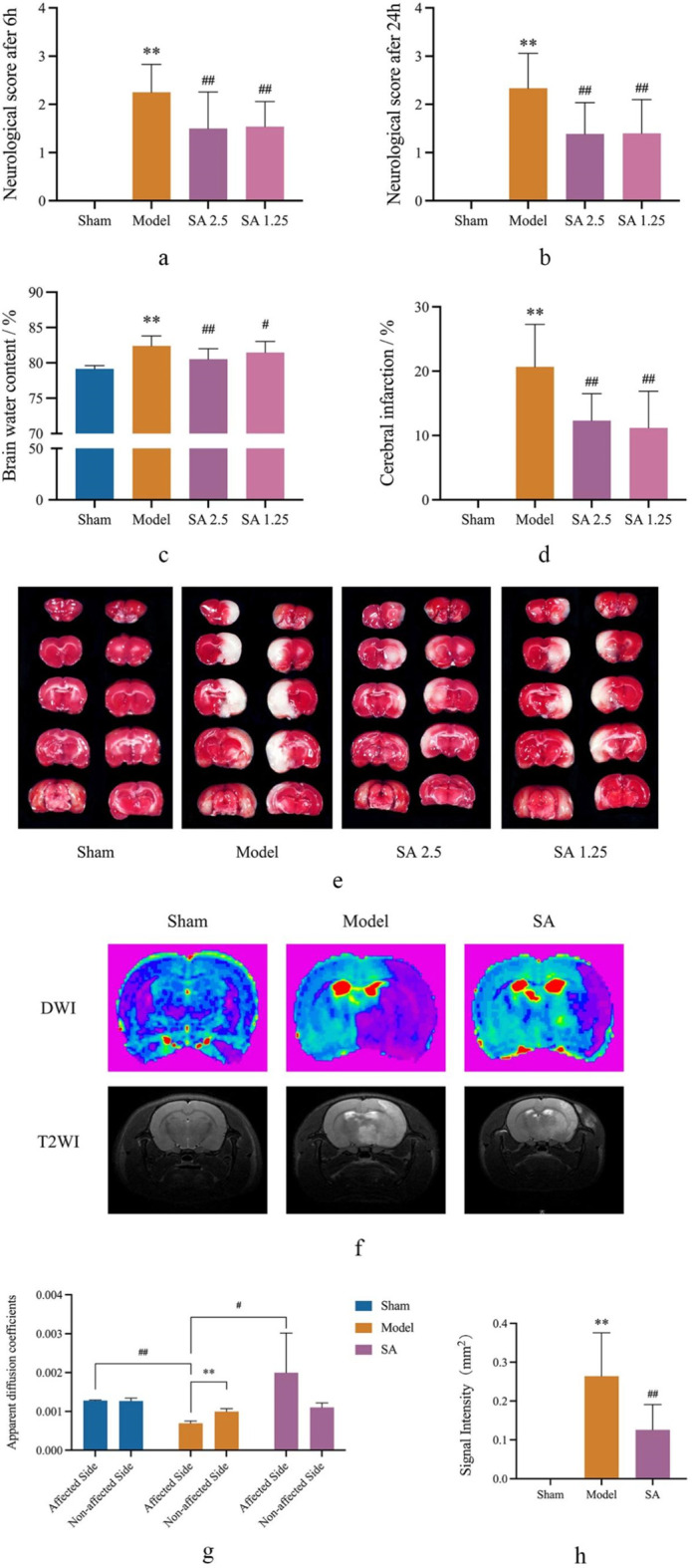
SA improves brain damage caused by CI. **(A, B)** Neurological score at 6 h and 24 h. **(C)** Cerebral water content. **(D, E)** Cerebral infarction. **(F–H)** DWI and T2WI in MRI scan. Data are expressed as mean ± SEM (n = 8).*p < 0.05 and **p < 0.01 vs. sham group; ^#^ *p* < 0.05 and ^##^ *p* < 0.01 vs. model group.

The results of neurobehavioral scores showed that compared with the Sham group, the neurobehavioral scores of rats in the Model group (6 and 24 h) were significantly higher than those in the Sham group (*p* < 0.01). Moreover, compared with the Model group, the neurobehavioral scores (6 and 24 h) of the high-dose (SA 1.25) and low-dose (SA 2.5) SA groups were significantly lower than those of the Model group (*p* < 0.01), as shown in Figures 2A, B.

The results showed that compared with the Sham group, the brain water content of the Model group was significantly larger than that of the Sham group (*p* < 0.01). Compared with rats in the Model group, both high and low doses of SA could reduce the brain water content of rats, and the difference was statistically significant (*p* < 0.05), as shown in Figure 2C.

TTC staining showed obvious infarct after 24 h of cerebral ischemia. Compared with the Sham group, the infarct area in the model group was significantly larger than that in the Sham group (*p* < 0.01). Compared with the Model group, both the SA 2.5 and SA 1.25 groups could reduce the cerebral infarction size of rats, and the difference was statistically significant (*p* < 0.01), as shown in Figures 2D, E. We then conducted more specific observations using MRI. The results indicated that in the SA group, the ADC value of the affected side of the brain was significantly larger than that in the Model group (*p* < 0.05), while the T2 signal was significantly reduced (*p* < 0.01). This further suggested that SA could improve infarct size and water dispersion after MCAO, as shown in Figures 2F–H.

The above efficacy experiment results showed that both high and low doses of SA had good therapeutic effects on cerebral ischemia reperfusion injury in rats, among which the SA 2.5 group had better effects on neurobehavioral scores, brain water content, and cerebral infarction size. Therefore, the high-dose SA group was used as the administration group for further discussion in the subsequent mechanism study.

### 3.2 Salidroside improves microstructural changes in the cerebral cortex caused by cerebral ischemia

**FIGURE 3:**
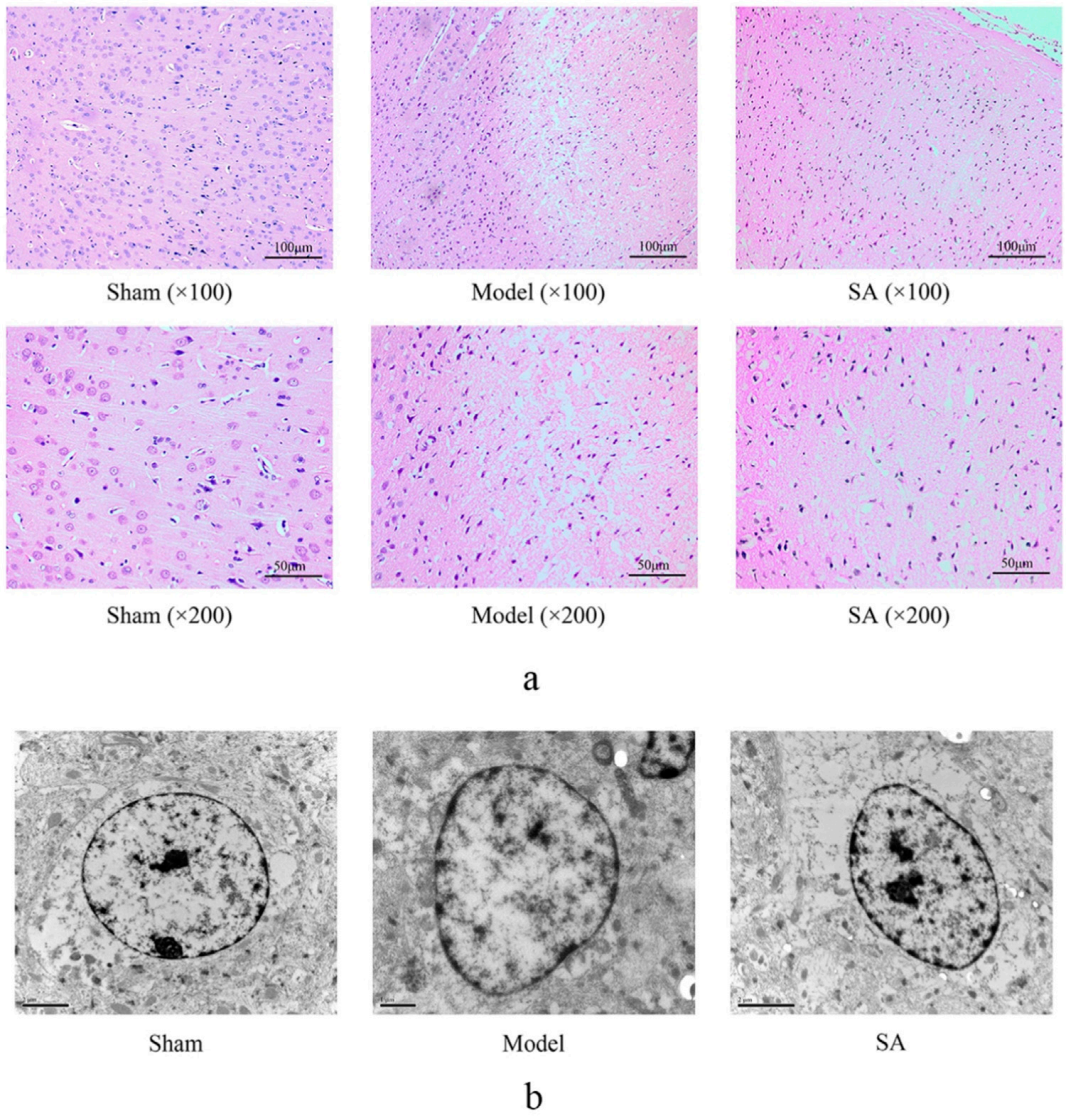
SA improves the microstructural changes in the cerebral cortex caused by CI. **(A)** HE staining of the brain cortex. **(B)** Astrocytes in the cerebral cortex were observed by transmission electron microscopy.

HE staining showed that no pathological changes were observed in the cerebral cortex of rats in the Sham group. The tissue arrangement was tight, the cell arrangement was clear, the morphology of the neurons was normal, and the structure was intact. In the Model group, the cerebral cortex of rats showed obvious pathological injury, cell arrangement was loose and disordered, cell necrosis was common, the number of neurons was reduced, and nucleus shrinkage and color were deepened. Compared with the Model group, the lesions in the SA group were reduced, the number of cortical neurons increased, the degree of nuclear shrinkage decreased, and the tissue arrangement was more complete and compact, as shown in Figure 3A.

The astrocyte cytoplasm is characterized by dispersed organelles and fibers. In the Model group, swollen astrocytes adhered to the damaged area, and a large amount of chromatin condensation was observed on the top of the nuclear membrane and the large nucleolus. The astrocyte damage was significantly improved in the SA group, as shown in Figure 3B.

### 3.3 Salidroside reduces the proliferation of astrocytes and the increase in GFAP expression in the cerebral cortex after cerebral ischemia

**FIGURE 4:**
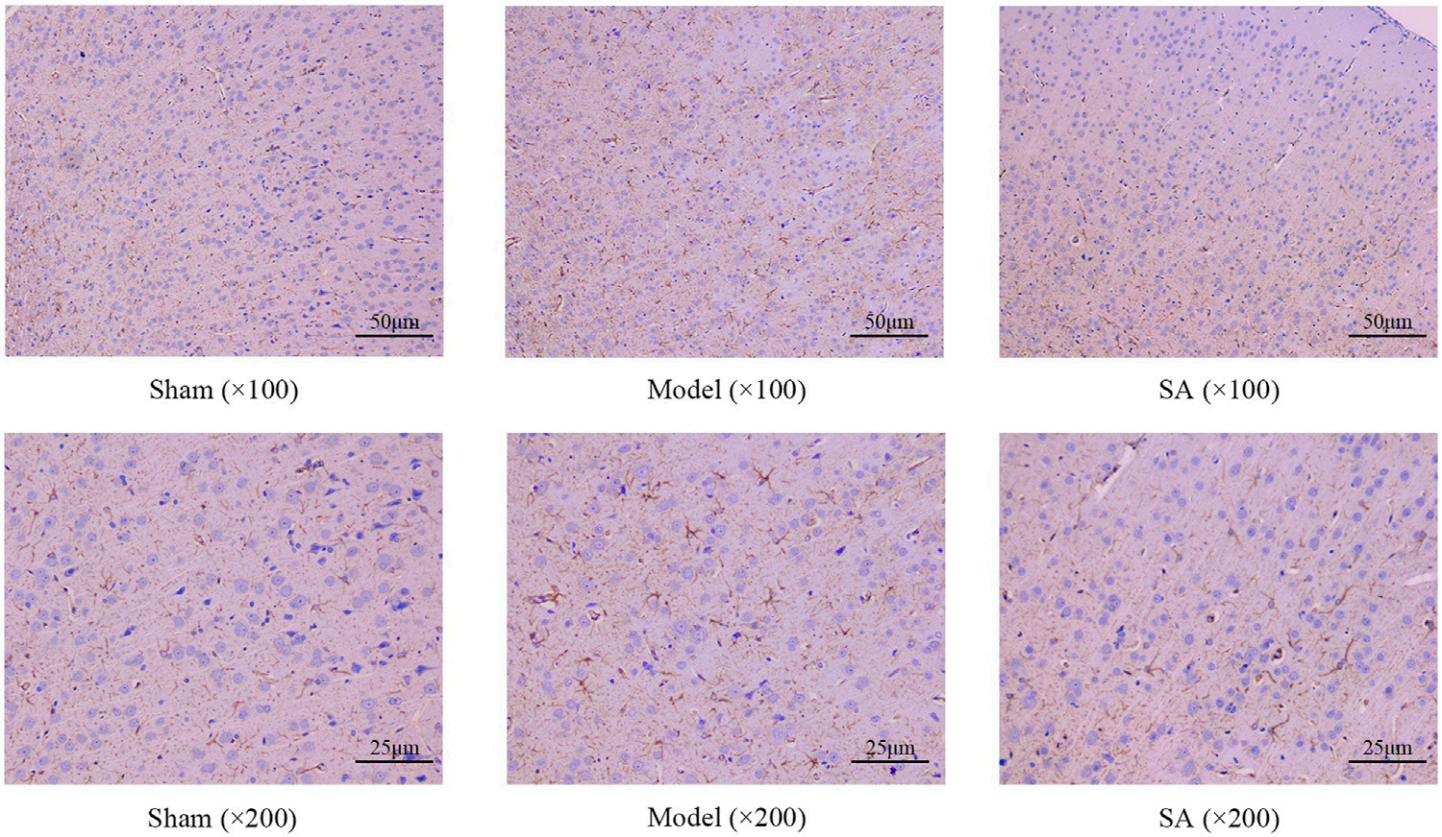
SA reduces the proliferation of astrocytes and increases GFAP expression in the cerebral cortex after CI.

Immunohistochemical staining showed that representative visual fields were selected in each group. In the Sham group, the astrocytes indicated by GFAP staining were evenly distributed and normal in shape. Compared with the Sham group, the number of astrocytes in the Model group, as shown by GFAP staining, was increased, the color was deepened, and the cell morphology was significantly swollen, indicating that there was a large number and obvious proliferation of astrocytes in the cerebral cortex of rats after cerebral ischemia. Compared with the Model group, the salidroside administration group of GFAP showed a decrease in the number of coloring and the degree of cell swelling, which significantly improved cell swelling and proliferation, as shown in Figure 4.

### 3.4 Salidroside improves SVGp12 cell damage induced by glucose deprivation

**FIGURE 5:**
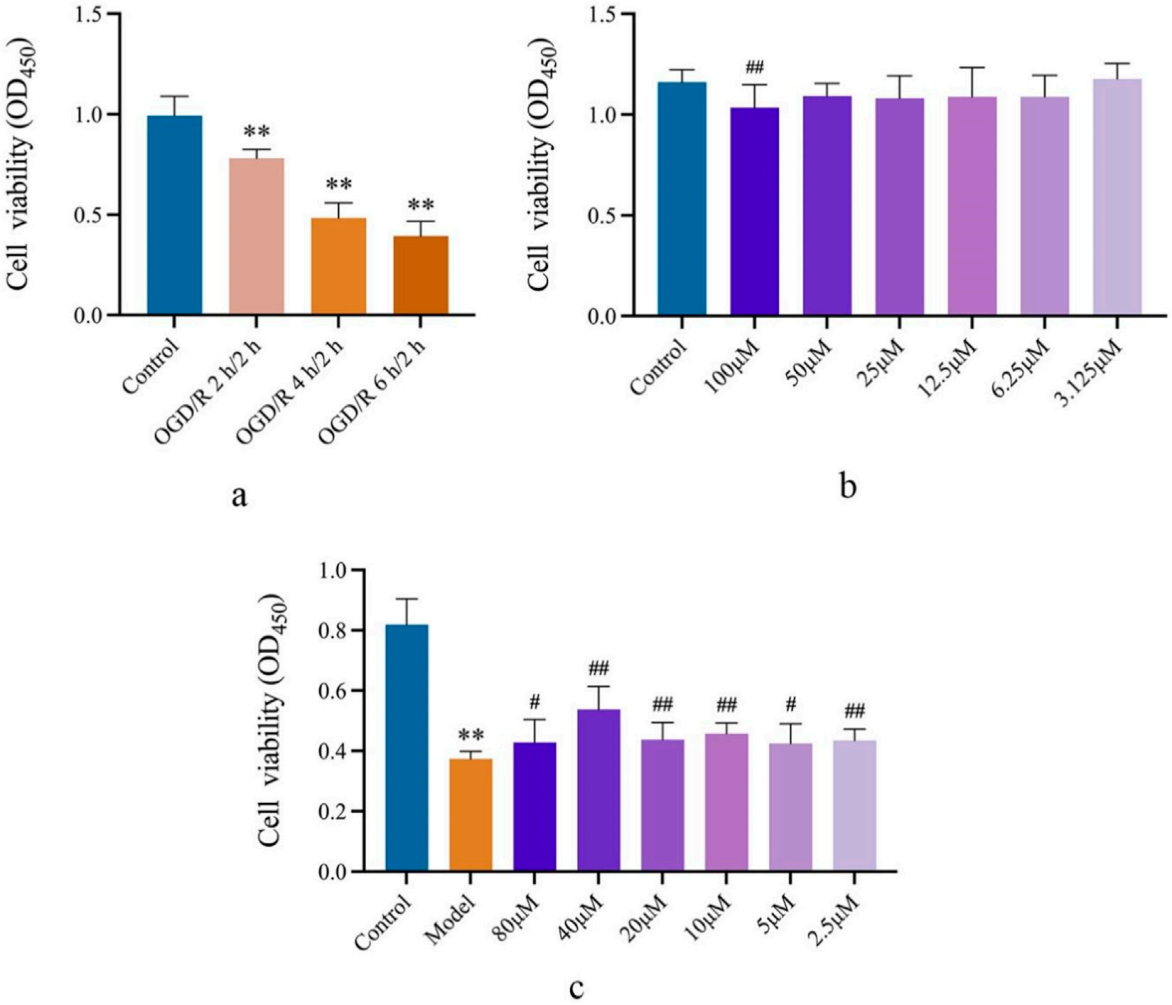
SA improves SVGp12 cell damage induced by OGD/R. **(A)** Determination of the OGD/R model of SVGp12 cells. **(B)** In the range of SA 50 μM and lower, the viability of normal SVGp12 cells was not affected. **(C)** SVGp12 cell viability was the highest when the SA was 40 μM. Data are expressed as mean ± SEM (n = 6).*p < 0.05 and **p < 0.01 vs. sham group; ^#^ *p* < 0.05 and ^##^ *p* < 0.01 vs. model group.

The effects of OGD/R on SVGp12 cell viability were investigated under OGD/R 2 h/2 h, 4 h/2 h, and 6 h/2 h conditions. The results showed that compared with the Control group, all three conditions could significantly reduce the cell viability of the cells (*p* < 0.01). Under the condition of OGD/R (4 h/2 h), the cell viability of SVGp12 cells was reduced by approximately 50%, so the condition of the subsequent cell model was OGD/R (4 h/2 h), as shown in Figure 5A.

In the selected safe concentration, SA showed no effect on normally cultured SVGp12 cells in the range below 50 μmol·L^−1^ compared to the Control group, as shown in Figure 5B.

In the pharmacodynamic test, compared with the Control group, the cell viability of SVGp12 cells in the Model group was significantly decreased (*p* < 0.01), and the SA group had a significant improvement in SVGp12 cells injured by glucose deprivation in the range of 2.5–80 μmol·L^−1^ (*p* < 0.05), and the cell viability was the best at 40 μmol·L^−1^. Subsequently, a dose of 40 μmol·L^−1^ will be selected to explore the mechanism, as shown in Figure 5C.

### 3.5 Salidroside reduces Glu content and increases GSH content

**FIGURE 6:**
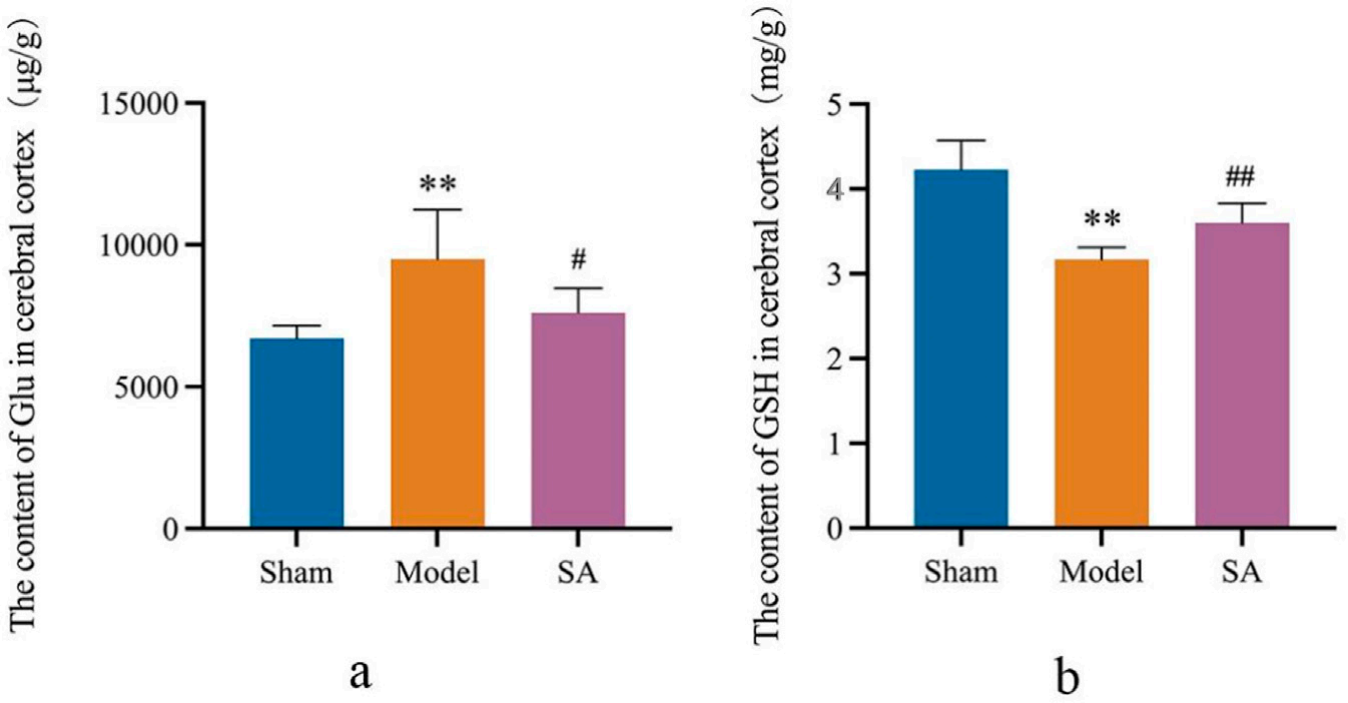
Salidroside reduces Glu content and increases GSH content in the cerebral cortex after CI. **(A)** Content of Glu in the cerebral cortex. **(B)** Content of GSH in the cerebral cortex. Data are expressed as mean ± SEM (n = 6). *p < 0.05 and **p < 0.01 vs. sham group; ^#^ *p* < 0.05 and ^##^ *p* < 0.01 vs. model group.

The results showed that after 24 h of reperfusion, the Glu and GSH content of brain tissue were significantly increased and decreased in the Model group compared with the Sham group (*p* < 0.01). Compared with the Model group, the Glu content and GSH content in the brain tissue of rats in the SA group were significantly decreased and significantly increased (*p* < 0.05), as shown in Figures 6A, B.

### 3.6 Salidroside improves the damage caused by cerebral ischemia by regulating GS and GLT-1 in the cerebral cortex and SVGp12 cells

**FIGURE 7:**
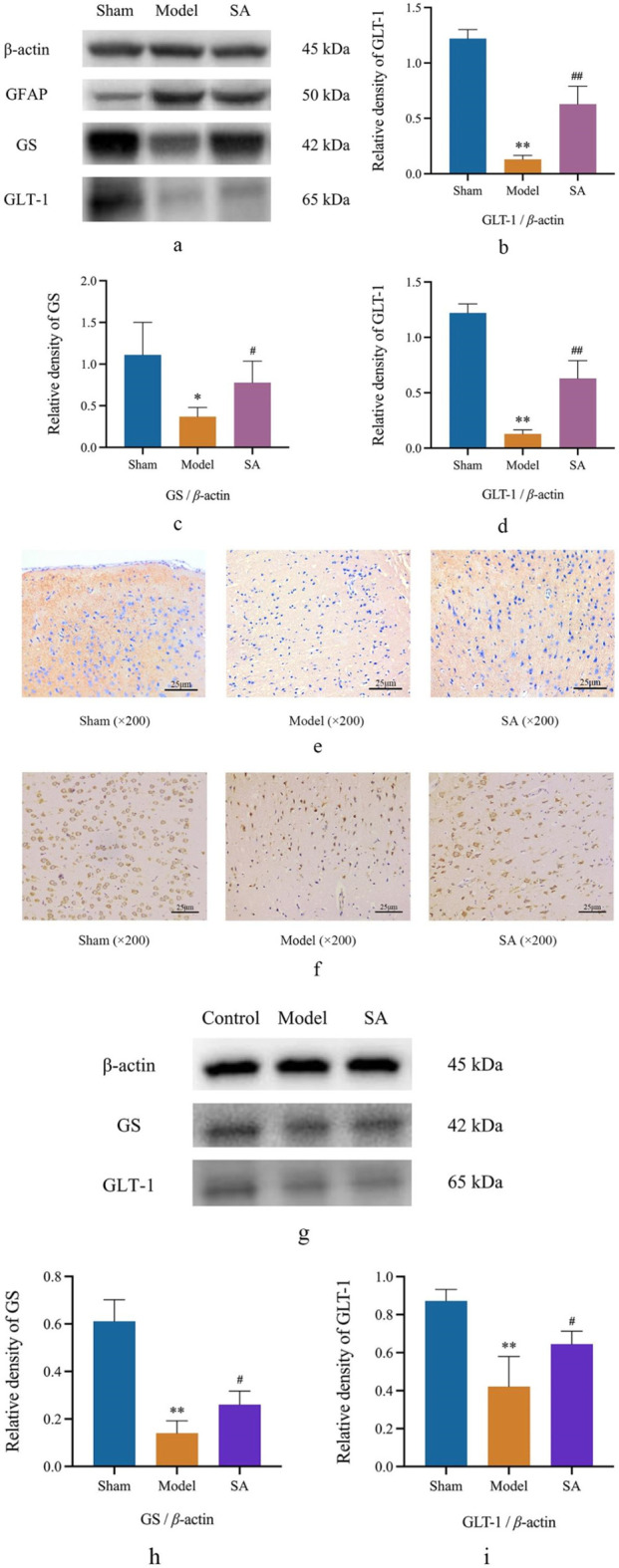
SA improves the damage caused by CI by regulating GS and GLT-1 in the cerebral cortex and SVGp12 cells. **(A–D)** Expression of GFAP, GS, and GLT-1 in brain tissue was detected by Western blot; **(E, F)** the expression of GS and GLT-1 was detected by immunohistochemistry; **(G–I)** the expression of GS and GLT-1 in SVGp12 was detected by Western blot. Data are expressed as mean ± SEM (n = 6). *p < 0.05 and **p < 0.01 vs. sham group; ^#^ *p* < 0.05 and ^##^ *p* < 0.01 vs. model group.

Western blot and immunohistochemical staining were used to determine the expression of GS and GLT-1 in the cerebral cortex and SVGp12 cells. After 24 h of cerebral ischemia, the expression of GS and GLT-1 in the cerebral cortex was significantly decreased (*p* < 0.01), and the expression of GS and GLT-1 in the cerebral cortex was significantly increased after high-dose administration of SA (*p* < 0.05). After OGD/R (4 h/2 h) in SVGp12 cells, the expression of GS and GLT-1 in SVGp12 cells was significantly decreased (*p* < 0.01), and the expression of GS and GLT-1 in the SA group was significantly increased (*p* < 0.05), as shown in Figures 7A–I.

## 4 Discussion

In this study, from the perspective of TCM, the main active ingredient of Rhodiola, salidroside, was selected to treat cerebral ischemic injury, and we focused on the brain edema caused by ischemia. First, the study observed the effects of SA on neurobehavior, brain water content, cerebral infarct size, and micromorphology of brain tissue in rats after MCAO and found that SA can improve the function of cerebral ischemic injury in MCAO rats. Next, the effects of SA on the morphology of astrocytes in brain tissue were observed by electron microscopy and immunostaining, focusing on the effects on cell swelling and cytotoxicity. Finally, the effect of SA on the activity of human astrocytes was observed by *in vitro* experiments, and it was verified that SA could affect the glutamate metabolic pathway on astrocytes at both the global and cellular levels, thereby improving cerebral ischemic injury.

Rhodiola has a long history and wide application in TCM theory and clinical use. It has always been known for its excellent pharmacological properties and the effect of “tonifying Qi and activating blood,” which is widely used in TCM prescriptions. First, SA is believed to improve the function of blood vessels. Studies have shown that SA can promote blood circulation (Yan et al., 2015), improve oxygen supply (Jiang et al., 2022), resist fatigue (Luo et al., 2019), and contribute to the recovery of damaged tissues by dilating blood vessels (Alameddine et al., 2015), especially in the case of cerebral ischemia. Second, SA is widely recognized as having antioxidant and anti-inflammatory properties (Zhu et al., 2016; You et al., 2021). Oxidative stress and inflammation are some of the main causes of neuronal injury induced by cerebral ischemia, and the antioxidant and anti-inflammatory properties of SA can reduce such damage and help protect neurons. In conclusion, SA is regarded as a potential natural pharmaceutical ingredient for the treatment of cerebral ischemic injury, and many reports with definite efficacy have been published in the research on the treatment of cerebral ischemia (Zhu et al., 2016; Jiang et al., 2022; Yan et al., 2015). In this study, we aimed to further explore the mechanism of SA, especially its regulation of the glutamate metabolic pathway in astrocytes, focusing on the regulatory effects of SA on GS and GLT-1, in order to provide more in-depth theoretical support for the treatment of cerebral ischemia.

Astrocytes are the most common glial cells in the central nervous system and play a crucial role in cerebral ischemia. Astrocytes are multifunctional cells with a wide range of physiological and metabolic functions, capable of maintaining the stability and health of neurons (Thrane et al., 2014). Astrocytes play a variety of key roles in the complex pathological process of cerebral ischemia, helping to mitigate neuronal damage. (a) Maintenance of the BBB (Díaz-Castro et al., 2023; Bergmann et al., 2018): The BBB is a highly selective biological barrier, and during cerebral ischemia, astrocytes can maintain the integrity of the BBB through tight connections, which helps to prevent fluid in the blood from entering the brain tissue. (b) Clearance of excitatory amino acids (Lee et al., 2022; Mahmoud et al., 2019): Cerebral ischemia leads to a significant increase in intracellular Glu levels. Glu is an excitatory neurotransmitter, and its excessive release and accumulation can lead to neuronal toxicity, which is one of the initial triggers of ischemic injury. GLT-1, also known as EAAT2, is expressed in astrocytes and is the molecule primarily responsible for clearing Glu from the cell. By efficiently taking up and clearing extracellular Glu, astrocytes help maintain the balance of excitatory substances in the brain and prevent the overactivation of neurons, thereby reducing excitatory toxicity, which is also the focus of this study. (c) Regulation of cytotoxic brain edema (Karve et al., 2016; Liang et al., 2007; Xiao et al., 2023): Cerebral ischemia can cause cytotoxic brain edema, resulting in increased intracellular water and cell swelling. Astrocytes can fight against cytotoxic brain edema to a certain extent, help maintain neuronal stability, and reduce neuronal damage. (d) Inflammation regulation (Linnerbauer et al., 2020; Colombo and Farina, 2016): Cerebral ischemia triggers an inflammatory response, resulting in the release of cytokines and the production of inflammatory mediators. Astrocytes can inhibit the release of inflammatory mediators and reduce the damage of inflammatory responses to neurons. (e) Maintaining the stability of neurons (Colombo and Farina, 2016; Kugler et al., 2021): Astrocytes can protect neurons by maintaining the stability of the neural environment, providing neuronal support, participating in the integration of nerve signals, and effectively communicating with vascular endothelial cells to ensure the balance of the brain environment. Therefore, astrocytes play multiple key roles in cerebral ischemia, and understanding these functions of astrocytes is crucial to further explore the mechanism of SA in cerebral ischemia because SA may play a role in protecting brain injury caused by cerebral ischemia by regulating the function of astrocytes after cerebral ischemia.

Glu is the main excitatory neurotransmitter in the central nervous system, where it plays a key role in transmission between neurons. Under normal conditions, after the release of Glu by neurons, astrocytes rapidly recover Glu through high-affinity transporters, inhibit the aggregation of Glu in the synaptic gap, and timely terminate the neurotransmitter function of Glu, which not only ensures the smooth transmission of information, but also prevents excitatory toxicity caused by excessive Glu concentration (Martinez-Lozada et al., 2016). GLT-1 and GS, which are mainly located in astrocytes, play a key role in the process of reabsorption of Glu. It is necessary to ensure that Glu is cleared at a normal rate under the action of GLT-1 and GS. Their interaction is essential for maintaining the normal function of neurons and the health of brain tissue and is an important way to reduce excitatory toxicity. During cerebral ischemia, Glu accumulates excessively, triggering overactivation of neurons and leading to excitotoxicity. Astrocytes regulate excitotoxicity due to ischemia and maintain low levels of extracellular Glu.

Among the aforementioned, GS is an enzyme whose main function is to promote the synthesis of Glu. GS can convert excess Glu into glutamine, thereby reducing the concentration of extracellular Glu and helping prevent the occurrence of excitatory toxicity. GLT-1 is a transporter protein that is also highly expressed in astrocytes, and its role is to take up Glu from outside the cell into the astrocyte, thereby reducing excessive accumulation of Glu between neurons. The role of GS and GLT-1 is particularly critical in cerebral ischemia. Cerebral ischemia can lead to a significant increase in Glu levels, as ischemia causes a disruption in energy metabolism, reducing the Glu clearance function of astrocytes. In this case, GS and GLT-1 work together to maintain Glu balance. Under pathological conditions, reduced transport activity or expression of GLT-1 leads to increased extracellular Glu concentration, which may lead to excitatory neurotoxicity (Grewer et al., 2008). GS and GLT-1 are also expressed in other types of cells other than astrocytes, including neurons, but only small amounts of Glu are imported into these cells via these transporters (Akbar et al., 1997). In addition, Glu can be transported in a bidirectional manner under pathological conditions, which may result in a net transport of Glu from inside the cell to outside (Persson and Rönnbäck, 2012). Overexpression of GLT-1 can significantly reduce brain injury induced by hypoxia (Harvey et al., 2011).

In this article, we have delved into the mechanism of SA action in cerebral ischemia, with particular focus on its regulation of GS and GLT-1 expression in astrocytes. By exploring SA, astrocytes, and their relationship, we try to understand the pathological mechanism of cerebral ischemia and the role of SA in the treatment of brain edema caused by cerebral ischemia. Based on our findings, we summarized and prospected the protective effect of SA on cerebral ischemia. We suggest that SA may regulate the expression of GS and GLT-1 in astrocytes to accelerate the clearance of Glu after cerebral ischemia and inhibit the cytotoxicity caused by excessive accumulation of Glu. This mechanism provides new theoretical support for the treatment of cerebral ischemia and lays a solid foundation for further research on the pharmacological properties and application prospects of SA. In order to further prove that the treatment of cerebral ischemia by SA is indeed achieved through the regulation of the glutamate metabolic pathway, we will explore the therapeutic principle of SA-specific regulation of the metabolic pathway from the perspective of molecular mechanism in the future. This study provides a new theoretical basis for the treatment of cerebral ischemia and the application of TCM, and we hope that it can provide useful guidance for future basic research and clinical practice to improve the quality of life of cerebral ischemia patients.

## 5 Conclusion

In conclusion, this study found that SA can ameliorate cerebral ischemia-induced injury by acting on astrocytes, and this process may be achieved by regulating the glutamate metabolic pathway in astrocytes.

## 6 Statement

The plant name has been checked with “World Flora Online” (www.worldfloraonline.org).

## Data Availability

The original contributions presented in the study are included in the article/Supplementary Material; further inquiries can be directed to the corresponding authors.

## Abbreviations

ADC, apparent diffusion coefficient; BBB, blood–brain barrier; BSA, bovine serum albumin; DWI, diffusion-weighted imaging; FOV, field of view; GFAP, glial fibrillary acidic protein; GLT-1, glutamate transporter 1; Glu, glutamate; GS, glutamine synthetase; GSH, glutathione; HE, hematoxylin–eosin; MCAO, middle cerebral artery occlusion; MRI, magnetic resonance imaging; MTX, matrix size; OGD/R, oxygen–glucose deprivation/reoxygenation; PVDF, polyvinylidene difluoride; SA, salidroside; SD, Sprague–Dawley; ST, slice thickness; T2WI, T2-weighted imaging; TCM, traditional Chinese medicine; TE, echo time; TR, repetition time; TTC, 2,3,5-triphenyltetrazolium chloride.
